# Letter to the Editor

**DOI:** 10.1186/s43058-022-00266-6

**Published:** 2022-02-11

**Authors:** Jean Yoon

**Affiliations:** 1 Health Economics Resource Center, VA Palo Alto Health Care System, 795 Willow Rd, 152-MPD, Menlo Park, CA 94025 USA; 2 HSR&D Center for Innovation to Implementation, VA Palo Alto Health Care System, Menlo Park, CA USA; 3 Department of General Internal Medicine, UCSF School of Medicine, San Francisco, CA USA

To the Editor:

Economic evaluations of implementation strategies remain few and far between despite the importance of economic considerations in disseminating implementation strategies. The piece “Moving Beyond Aim 3” addresses this gap by presenting a model of transdisciplinary team science for implementation scientists and health economists that can increase economic evaluations. As the authors state, health economists are often approached to conduct “aim 3” of an implementation study on the costs of implementation; however, “aim 3” is rarely considered a high priority as reflected by the study resources devoted to that aim. The authors argue that transdisciplinary science moves beyond traditional collaborations across disciplines that combine methods and theories from different disciplines in a hybrid approach.

In this transdisciplinary team science model, the development phase occurs when researchers from different disciplines agree to collaborate and develop a shared understanding of the problem and of the goals of the group. In this phase, the authors highlight the challenges in collaborating with health economists due to the priority of health economists publishing in economic journals and their lack of priority around collaboration. However, this description of health economists focuses on those trained in and/or employed by departments of economics whereas health economists with expertise in evaluating applied health care interventions and who have an orientation toward collaboration across disciplines are often trained in and/or employed by multidisciplinary departments in schools of public health, public policy, medicine, and pharmacy. As such, implementation scientists may have more success seeking out health economists who do not have a single-discipline focus. As the authors suggest, it is important to identify health economists with interests in implementation science, and they highlight Centers for Translational Science Award Programs as places to find health economists.

The authors also describe a phase in their team science model, the conceptualization of research questions and designs by the study team in which the integration of multiple disciplines occurs. The authors further discuss participation of health economists in evaluating implementation costs and other economic outcomes such as quality of life. What could be added to the description of the conceptualization phase, however, is the often-overlooked expertise of health economists in quantitative methods to answer the central aims of whether an implementation strategy is effective. While some implementation studies employ a randomized design to estimate the unbiased effect of implementation strategies versus usual care on outcomes, many more use nonrandomized designs and retrospective analysis of observational data. Many health economists have expertise in econometric methods to estimate the causal impact of interventions or programs. Methods such as differences-in-differences, instrumental variables, and propensity matching involve econometric or statistical techniques to limit bias in non-randomized studies comparing outcomes between intervention and control groups since these groups can have underlying differences that lead to misattributing differences in outcomes to the intervention. For example, one implementation study evaluated the impact of a quality improvement program for maternal and newborn care on newborn outcomes for earlier versus later-adopting hospitals using a differences-in-differences design to compare outcomes before and after the program was implemented in the two groups of hospitals since hospitals adopting the program are more similar to each other than to hospitals who never adopted the program [1]. Another implementation study compared use of internal and external facilitation to implement a nurse-led intervention on transitions in care and used propensity-matched facilities to identify a comparison group of facilities who did not implement the intervention to compare patient outcomes between intervention and comparison facilities [2]. In these and many more examples, the study designs and statistical methods allowed for estimating the impact of implementation interventions on outcomes by reducing the bias created when hospitals, providers, or patient groups receiving interventions have many differences with those not receiving interventions. Therefore, health economists need not be constrained to “aim 3” when their expertise could be integrated into helping answer key implementation science questions.

Overall, the authors present reasonable approaches towards fostering better collaborations between health economists and implementation scientists in multidisciplinary teams. Key questions around costs and resource use of implementation strategies and their downstream impacts on health system costs and other economic outcomes continue to be important to informing the diffusion of real-world implementation strategies. Whether adoption of this model by multidisciplinary teams leads to greater inclusion of economists and economic evaluations in implementation science remains to be seen. Limited resources for conducting research will always lead to prioritizing research questions, and consensus must still be obtained on the inclusion of economic outcomes relative to other study outcomes. Efforts to increase economic evaluation can be bolstered by health care organizations, policymakers, and journals asking for more information on economic implications of implementation strategies. As the evidence base of implementation science continues to grow, building capacity for economic evaluations is critical.

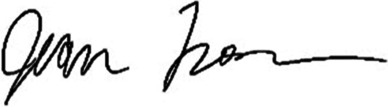



Jean Yoon, PhD, MHS

## Data Availability

Not applicable.
